# Application of Metabolomics to Study Effects of Bariatric Surgery

**DOI:** 10.1155/2018/6270875

**Published:** 2018-03-11

**Authors:** Paulina Samczuk, Michal Ciborowski, Adam Kretowski

**Affiliations:** ^1^Clinical Research Centre, Medical University of Białystok, Białystok, Poland; ^2^Department of Endocrinology, Diabetology and Internal Medicine, Medical University of Białystok, Białystok, Poland

## Abstract

Bariatric surgery was born in the 1950s at the University of Minnesota. From this time, it continues to evolve and, by the same token, gives new or better possibilities to treat not only obesity but also associated comorbidities. Metabolomics is also a relatively young science discipline, and similarly, it shows great potential for the comprehensive study of the dynamic alterations of the metabolome. It has been widely used in medicine, biology studies, biomarker discovery, and prognostic evaluations. Currently, several dozen metabolomics studies were performed to study the effects of bariatric surgery. LC-MS and NMR are the most frequently used techniques to study main effects of RYGB or SG. Research has yield many interesting results involving not only clinical parameters but also molecular modulations. Detected changes pertain to amino acid, lipids, carbohydrates, or gut microbiota alterations. It proves that including bariatric surgery to metabolic surgery is warranted. However, many molecular modulations after those procedures remain unexplained. Therefore, application of metabolomics to study this field seems to be a proper solution. New findings can suggest new directions of surgery technics modifications, contribute to broadening knowledge about obesity and diseases related to it, and perhaps develop nonsurgical methods of treatment in the future.

## 1. Introduction

Based on historical reports, origins of bariatric surgery date back to the 10th century, when King of León, Sancho I (called the Fat), was treated by famous Jewish doctor, Hasdai ibn Shaprut [1]. The homeland of bariatric surgery in modern meaning is the University of Minnesota. The first metabolic surgery was the jejunoileal bypass performed there by Arnold J. Kremen in 1954 [1, 2]. Nevertheless, success has many parents; therefore, many great minds were involved in the bariatric surgery development. As the pioneers in this medicine field, Henry Buchwald, Richard L. Varco, Edward E. Manson (long recognized as the father of bariatric surgery), Allan C. Wittgrove, Nicola Scopinaro, Walter J. Pories, Picard Marceau, and Douglas S. Hess have to be mentioned [1, 3]. Of course, they are a small group from all people developing bariatric surgery discipline, but their contributions to this area are incontrovertible.

Bariatric surgery has come a long way since the first procedures. Its evolution can be seen from several perspectives. Henry Buchwald spotlights the fact that bariatric surgery is metabolic surgery. In 1978, he and Richard L. Varco published a book entitled *Metabolic Surgery* in which they defined it as “the operative manipulation of a normal organ system to achieve a biological result for a potential health gain.” Therefore, in the early beginnings of bariatric surgery, not only weight loss but also general health improvement was already expected. Other years carried out other studies, and new reports about benefits are evoked by bariatric procedures. Toward the end of the previous century, it was communicated that bariatric surgery can resolve type 2 diabetes mellitus. What is really interesting is that the remission of T2DM can occur very fast, even before the patients can reduce their weight [4–6]. It has also been pointed out that bariatric procedures improve many other clinical parameters (BMI, HbA1c, glucose and cholesterol levels, insulin resistance, and modulations of gut hormones) [7]. However, those modulations are not the core of this paper. Evidently, like every surgical intervention, it is related to some risks and disadvantages. Depending on the type of surgery, patients can be exposed to, for example, dumping syndrome or be forced to lifetime vitamin supplementation [6]. However, the growth of laparoscopic surgery with its reduced complications such as wound infection, incisional hernias, and lower early postoperative morbidity and mortality resulted in shorter hospital stay, faster recovery, lower morbidity, and improved effects, which has led to an ever-increasing patient demand [1, 8, 9]. Therefore, bariatric surgery has become the most effective treatment of morbid obesity and associated comorbidities, such as sleep apnea, hypertension, dyslipidaemia, and type 2 diabetes [8, 10, 11]. Thus, these kinds of surgical procedures not only reduce overall mortality but also improve patients' quality of life [9, 12].

The history of bariatric surgery is dominated by six procedures, which are jejunoileal bypass (JIB), Roux-en-Y gastric bypass (RYGB), vertical-banded gastroplasty (VBG), biliopancreatic diversion (BPD) or its familiar duodenal switch (DS), adjustable gastric banding (AGB), and sleeve gastrectomy (SG) [3, 6]. Nowadays, Roux-en-Y gastric bypass and sleeve gastrectomy are considered as the “gold standard” bariatric interventions [9, 13–16].

Similarly to those of bariatric surgery, the origins of metabolomics can be found far back in the past—in ancient Greece. Likewise, the beginning of metabolomics in modern meaning is estimated to be in the 1960s, when during metabolic-control analysis, the mathematical method for cell metabolism modelling was developed. The second starting point was the development of nuclear magnetic resonance (NMR) spectroscopy [17]. Currently, Oliver Fiehn and Jeremy K. Nicholson are considered as pioneers in the metabolomics (metabonomics) field [18–20]. Nowadays, both terms are used interchangeably. Jeremy K. Nicholson described differences between these terms as philosophical rather than technical. Metabolomics looks for an analytical description of complex biological samples. Its aim is to characterize and quantify all the small molecules in the studied sample. In the meantime, metabonomics is described as global measurement of dynamic metabolic response of living systems to biological stimuli or genetic manipulation. It is focused on understanding systemic modulations of complex multicellular systems through the time. Actually, modelling procedures for both of them are the same [17]. Metabolomics analyses are based on stand-alone hydrogen nuclear magnetic resonance (^1^H NMR) technique or mass spectrometry technique combined with different metabolite chromatographic separation methods, that is, liquid chromatography (LC), gas chromatography (GC), or capillary electrophoresis (CE). This range of analytical platforms enable detection, characterization, and quantification of low-molecular-weight metabolites from different classes, for example, lipids, amino acids, peptides, nucleic acids, organic acids, vitamins, thiols, carbohydrates, and many other metabolites in which mentioned species can be metabolised. NMR can uniquely identify and simultaneously quantify a wide range of organic compounds in the micromolar range. It has been used for analysis of amino acids, nucleotides and nucleosides, vitamins, thiols, carbohydrates, and peptides. The LC-MS method has become a useful tool for the analysis of hundreds of polar metabolites in a complex sample. It is an important tool used for targeted or nontargeted metabolomics. Liquid chromatography separation is better suited for the analysis of labile and nonvolatile polar (hydrophilic interaction liquid chromatography (HILIC)) and nonpolar (reversed-phase chromatography) compounds in their native forms. Additionally, MS and LC are commonly used for compound characterization and to obtain structural information. GC-MS has been used as a platform especially for hydrophilic metabolites. Using this approach, one can directly separate and quantify the volatile metabolites. It allows to profile several hundreds of compounds including organic acids, most amino acids, sugars, sugar alcohols, aromatic amines, and fatty acids. CE-MS has been used for both targeted and nontargeted analyses of polar and ionic metabolites, including analysis of inorganic ions, organic acids, amino acids, nucleotides and nucleosides, vitamins, thiols, carbohydrates, and peptides [21, 22].

Therefore, metabolomics is a powerful tool for the comprehensive study of the dynamic alterations of the metabolome. It has been widely used in the areas of medicine, biology, and physiology for biomarker discovery or for prognostic evaluations [23–27].

The aim of this study was to find and classify all studies in which the metabolomics approach was used to study the metabolic effects of bariatric surgery published up till now. Additionally, detected metabolites were investigated together to obtain conclusions about the impact of bariatric surgery on particular biochemical pathways.

## 2. Methods

### 2.1. Data Source and Study Selection

PubMed was searched for keywords such as metabolomics, bariatric surgery, LC-MS, GC-MS, CE-MS, NMR, LSG, and RYGB. The last search was performed in July 24, 2017, and only publications up to this date are included. Additionally, results were limited to papers written in the English language. All studies, regardless of species (humans, rats, and mice) and biological samples used (blood, urine, and tissues), were included in this review. Intervention trials in which metabolomics techniques were used to study changes after bariatric surgery were investigated. In one study, influence of diet and surgery was compared, but only blood taken during bariatric procedure was analyzed [28]. This study (and animal studies) is reviewed, but its results were not considered during the MetaboAnalyst analysis.

### 2.2. Metabolomics Data Analysis with MetaboAnalyst 3.0

MetaboAnalyst 3.0 was used to perform biochemical interpretation of all altered metabolites. This online tool (http://www.metaboanalyst.ca/) allows to analyze impact of particular compounds on biochemical pathways. In MetaboAnalyst 3.0, there are currently 15 pathway libraries supported, with a total of 1173 pathways (80 for *Homo sapiens*). The pathway analysis module combines results from the powerful pathway enrichment analysis with those from the pathway topology analysis. Pathway analysis accepts a list of compound labels (common names, HMDB IDs, or KEGG IDs). Next, Fisher's exact test or hypergeometric test is used. The results from the pathway analysis are presented graphically as well as in a detailed table [29].

## 3. Results

**Figure 1:**
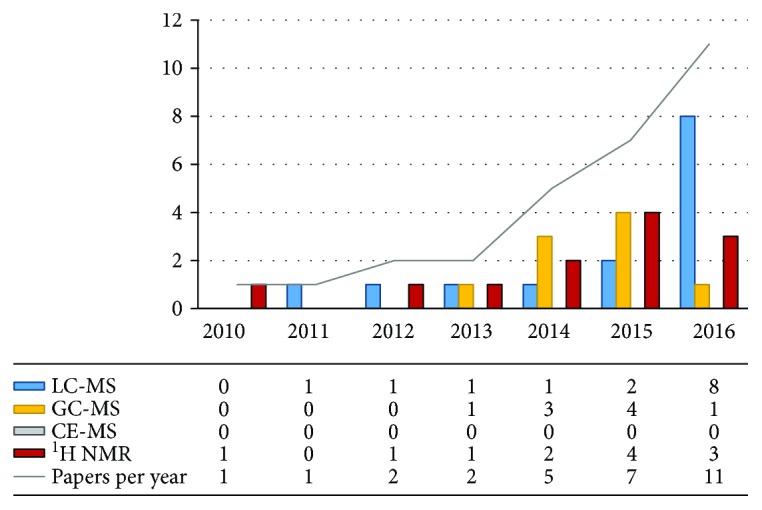
Summary of published studies.

Of all initially retrieved studies, 30 successfully fit the criteria for this review. The first study on the application of NMR to examine effects of biliopancreatic diversion and Roux-en-Y gastric bypass was published in 2010. Since then, the number of papers per year is still rising (Figure 1). Additionally, also other metabolomics platforms were used to investigate the influence of different types of surgeries on metabolome. This proves the growing interest on bariatric surgery and applicability of metabolomics to investigate this metabolic surgery.

Among all examined procedures [7, 11, 13, 25, 26, 28, 30–39], Roux-en-Y gastric bypass comprised over half (53.7%). The second most studied was sleeve gastrectomy (29.3%) [7, 26, 27, 30, 31, 38–44]. Investigations on other techniques—duodenal-jejunal endoluminal bypass (4.9%) [37, 45], laparoscopic gastric banding (4.9%) [7, 46], biliopancreatic diversion (4.9%) [47, 48], and duodenal-jejunal endoluminal bypass liner (2.4%) [49]—were definitely less often.

Liquid chromatography coupled with mass spectrometry [7, 11, 13, 25, 30–32, 41–44, 48, 50, 51] and hydrogen nuclear magnetic resonance [11, 26, 33, 35, 37–40, 45–47, 49] were the most commonly used analytical platforms (Figure 1, Table 1). Gas chromatography was also a rather commonly used technique [7, 27, 28, 34–37, 42, 44, 52]. Hitherto, capillary electrophoresis was not applied to study the effects of bariatric procedures. There was no domination of targeted (51%) or untargeted (49%) type of metabolomics analysis.

Most of the studies were focused on human samples; only few were performed on rats [11, 13, 28, 45] or mice [42]. In some of them, animal and human studies were combined [28, 42].

Serum (47%) [7, 25, 28, 31, 32, 38, 41–45, 48, 49, 51, 52] and plasma (25%) [11, 27, 30, 33, 34, 37] were the most commonly used biological materials. Other analyzed samples were urine (12%) [13, 26, 37, 39] and different types of tissues (16%)—heart [11], liver [40, 45], or adipose tissue [28, 50]. Interestingly, a study on atypical material (omental adipose tissue) was performed by García-Alonso et al. [50].

Most of the studies were focused on obesity, including morbid obesity, and type 2 diabetes. Interestingly, in one research, another disease, that is, nonalcoholic fatty liver disease (NAFLD), was examined [40]. Some of the studies were focused only on effects of bariatric surgery. In one interesting study, measurement of biological age was performed on a group of obese patients after bariatric surgery [26].

Usually, researches were performed on medium-sized groups, that is, 10–20 patients. There were also few studies on really small (below 10), big (30–50), or really big (up to 100) [38] groups of patients. For animal study standards, a really large group of rats (27 animals) was used to study RYGB surgery effects [11].

Considering time intervals, very different time points were examined. Most of the studies contained baseline and then one or more follow-up points. The shortest difference between baseline and the first analyzed time point (below 7 days after surgery) was presented by Jüllig et al. [27], Nemati et al. [30], Arora et al. [36], Gralka et al. [38], and Friedrich et al. [39]. The longest follow-up was presented in the study performed by Heffron—1, 2, 3, 4, and 5 years after the surgery [46].

**Figure 2:**
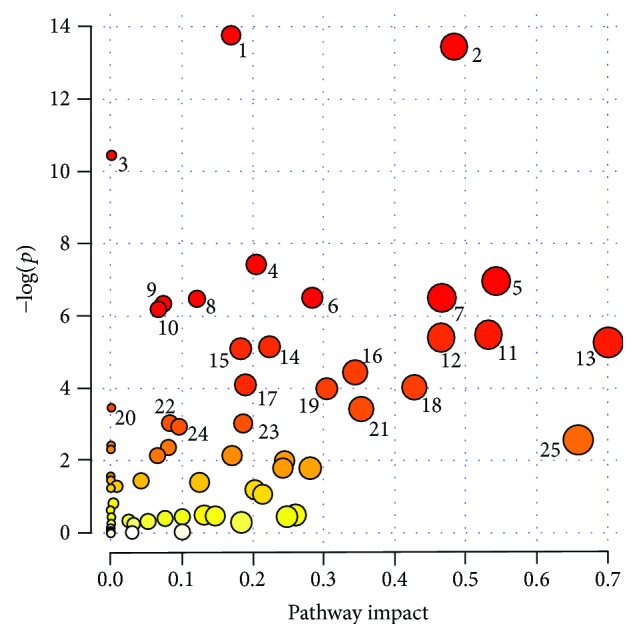
Summary of pathway analysis. The following are the top 25 identified statistically significant pathways: 1: aminoacyl-tRNA biosynthesis; 2: glycine, serine, and threonine metabolism; 3: nitrogen metabolism; 4: phenylalanine metabolism; 5: cysteine and methionine metabolism; 6: citrate cycle (TCA cycle); 7: taurine and hypotaurine metabolism; 8: valine, leucine, and isoleucine biosyntheses; 9: propanoate metabolism; 10: nicotinate and nicotinamide metabolism; 11: alanine, aspartate, and glutamate metabolism; 12: arginine and proline metabolism; 13: synthesis and degradation of ketone bodies; 14: pyrimidine metabolism; 15: methane metabolism; 16: glutathione metabolism; 17: glyoxylate and dicarboxylate metabolism; 18: pyruvate metabolism; 19: purine metabolism; 20: pantothenate and CoA biosyntheses; 21: d-glutamine and d-glutamate metabolism; 22: valine, leucine, and isoleucine degradation; 23: butanoate metabolism; 24: glycolysis or gluconeogenesis; 25: linoleic acid metabolism.

From examined studies, over 300 metabolites were selected. After removing duplicates, standardizing names (taking into account synonyms), and assigning HMDB IDs, we introduced 224 compounds into MetaboAnalyst 3.0 software. Finally, the software used 211 of them for pathway analysis. Obtained results showed that metabolites altered by bariatric surgery belong to 63 biochemical pathways. A statistically significant influence was exhibited for 23 of them. Based on the MetaboAnalyst results, the most impacted pathways after bariatric interventions are aminoacyl-tRNA biosynthesis; glycine, serine, and threonine metabolism; nitrogen metabolism; phenylalanine metabolism; cysteine and methionine metabolism; TCA cycle (citrate cycle); taurine and hypotaurine metabolism; valine, leucine, and isoleucine biosyntheses; propanoate metabolism; and nicotinate and nicotinamide metabolism (Figure 2).

All the above-described data are detailed in Tables 1 and 2.

## 4. Discussion

Despite the substantial research activities in the last years, many molecular aspects of bariatric surgery consequences leading to observed surgery effects are still unknown. However, alterations of particular metabolite groups detected till now allow to deduce about general changes associated with this kind of surgical procedure.

### 4.1. Amino Acid Alterations

One of the biggest group of metabolites altered by bariatrics procedures are amino acids (AA) [25]. Changes in the level of alanine [26, 27, 32, 38, 40, 42, 45], arginine [38], cysteine [45], glutamate [7, 33, 35, 40, 42], glutamine [11, 35, 38, 42, 43, 45], glycine [26, 32, 38, 39, 42, 43], histidine [26, 27, 38, 44], homocysteine [45], proline [36], lysine [11, 26, 40, 42], methionine [36, 41, 42, 45], ornithine [32], phenylalanine [7, 25, 27, 32, 34, 38, 40], proline [27], serine [42, 45], threonine [26, 27, 35], and tyrosine [25, 26, 38, 40–42] were observed. Among AA, the frequently modulated group was branched-chain amino acids (BCAA)—isoleucine [7, 25, 32, 33, 35, 36, 38, 40, 42, 45], leucine [7, 32, 34–36, 38, 40, 42], and valine [7, 25, 26, 32, 33, 35, 36, 38, 40, 42, 45]. Also small peptides such as glutathione [44, 45], amino acid derivatives [25], or products of their chemical modulations like phenylacetyl-l-glutamine (PAGN) [25] were found to be altered.

Higher serum concentrations of phenylalanine, tyrosine, leucine, isoleucine, valine, and glutamate were noticed in obese individuals. Roux-en-Y gastric bypass caused a decrease of circulating aromatic amino acids (AAA): methionine, alanine, and lysine. Serum concentrations of serine and glycine were found to be increased after sleeve gastrectomy [42]. RYGB accelerates caseinate digestion and amino acid absorption, resulting in a faster and higher but also more transient postprandial elevation of plasma amino acids [34]. In the group of patients with diabetes remission, relatively to nonremission significant decrease in alanine after one year was observed [32]. Branched-chain amino acid levels were found to be correlated with decreased insulin resistance [7].

### 4.2. Lipids Modulation

Another large group of metabolites modulated by bariatric procedures is lipids. Among them, alterations of phosphatidylcholines [11, 25, 30, 36, 41], lysophosphatidylcholines [11], phosphatidylethanolamines [7, 25, 40], lysophosphatidylethanolamines [11], phosphatidylinositol [40], sphingomyelins (SM) [25, 36, 41], cholesterol and its fractions [33, 35, 46], triglycerides [36, 40], and monoacylglycerols [44] were observed. An important group of affected lipids is composed of the fatty acids (FA) [52], especially free fatty acids (FFA) [25] and their esters (FAME) [28]. Modulations of monounsaturated (MUFAs) [44] and polyunsaturated fatty acids (PUFAs) [30, 40] were also described. Alterations in the levels of palmitoleic acid [11, 30], eicosadienoic acid [51], linoleic acid [30, 40, 51], stearic acid [30, 36, 51], or palmitic acid [30, 36, 51] were highlighted.

Higher baseline stearic acid/palmitic acid ratio was associated with greater probability of diabetes remission after RYGB and may serve as a diagnostic marker in preoperative patient assessment. Correlation analysis demonstrated that the stearic acid/palmitic acid ratio negatively correlated with HbA1c, TG, TC, LDL-c, and HOMA-IR and positively correlated with HDL-c in overweight and obese subjects [51]. Arora et al. reported early alterations in the metabolome and lipidome after gastric bypass in insulin-resistant morbidly obese subjects. The beneficial effects of surgery included a reduction in BCAA metabolites and short-chain TGs [36]. Data obtained by Oberbach et al. showed that LSG affects the amino acid and lipid metabolism. It leads to modification of amino acids and lipid metabolism as indicated by changes in glycerol-phosphatidylcholines and SM levels [41].

### 4.3. Gut Microbiota-Related Metabolites

Gut microbiota plays an important role in various processes including energy metabolism, lipid accumulation, homeostasis, regulation of brain function, and behavior. Its modulation is also one of the mechanisms by which bariatric surgery promotes weight loss and type 2 diabetes remission [53]. The human gastrointestinal tract (GIT) is dominated by two bacterial phyla, the Bacteroidetes and the Firmicutes, and the proportion of this phyla to each other has been already linked to obesity and type 2 diabetes mellitus [53–56]. Accordingly, alterations of compounds which can be linked to gut microbes and their modulations by surgery are important and interesting findings [38]. In several studies, changes in levels of SCFAs (i.e., butyric acid) [26, 27, 37–39, 44, 47], lactate [7, 26, 33, 35, 45], indole [36], and 3-indoxyl sulfate [7, 26] after bariatric procedures were observed. Alterations in the level of sulfate-containing metabolites can be expected, as the largest group of sulfate-reducing bacteria is found among the Proteobacteria, present mainly in the duodenum, which is modified in some bariatric procedures [57]. Also levels of mentioned cholesterol [33, 35, 46], l-carnitine [7, 11, 26, 43], and niacin or choline [7, 25, 26, 32, 45] can be linked to gut microbes [58]. Modesitt et al. linked perturbations in tryptophan, phenylalanine, and heme metabolism with decreased inflammation and alterations in the intestinal microbiome [7]. Moreover, tyrosine or phenylalanine fermentation by intestinal bacteria generates *p*-cresol. *Bacteroides fragilis* is one of the bacteria that have been shown to produce it [59]. Serum glutamate concentration was inversely correlated with the abundance of some *Bacteroides* species as well [42]. Changes in histidine and its metabolites following surgery might be an indication of altered gut microbiome ecology or liver function [44]. Another significant metabolite in which its modification can be linked to microbiota modification is beta-hydroxybutyrate (*β*-OHB). It is derived mainly from the oxidation of fatty acids and is the first ketone produced in the fasting state. Additionally, it is also produced in the form of poly-*β*-OHB by prokaryotes when carbon sources are freely available but other nutrients are limited [60].

### 4.4. Other Compounds

Some of the metabolites reported in reviewed studies as significantly changing after bariatric surgery do not belong to the above-described groups of compounds. Therefore, in this section, metabolites from other biochemical pathways altered by metabolic surgery will be presented.

Examples of such compounds are 8-oxoGua, 8-oxoGuo, and 8-oxodG, markers of DNA and RNA damage studied by Bankoglu et al. [13]. Alterations in the concentration of these compounds indicate the association of obesity with increased oxidative stress and DNA damage. Moreover, it was said that RYGB or caloric restriction can significantly reduce elevated oxidative or nitrative stress as well as genomic damage in obese subjects. Results obtained by Sarosiek et al. also suggested that bariatric surgery might promote antioxidant defence and insulin sensitivity through both increased heme synthesis and heme oxygenase (HO) activity or expression [44]. Modification of nucleotide metabolism after bariatric intervention was evaluated by adenosine, inosine, hypoxanthine, xanthine, urate, and allantoin profiling [7]. DJB surgery enhanced trans-sulfuration and its consecutive reactions such as detoxification and the scavenging activities of reactive oxygen species [45]. Metabolites detected by Narath et al. (trimethylamine *N*-oxide, alanine, phenylalanine, and indoxyl sulfate) are known as cardiovascular disease risk markers [32].

An important group of altered metabolites is compounds connected with energetic processes. Pyruvate [7, 11, 38, 44, 45], citric acid [11, 26, 27, 38], carnitines [7, 11, 26, 43], or the above-mentioned fatty acids belong to this group. Calvo et al. observed that the presence of moderate NAFLD is common in young patients with morbid obesity. Their data may be useful to explain the dissociation between excess lipid storage in adipose tissue, NAFLD, and insulin resistance [40]. After surgeries, energy metabolism, glucose homeostasis, and glycemic markers showed marked improvements, which manifested with reduced levels of glucose and the glycolytic end products of pyruvate and lactate. An increased level of chiro-inositol may be associated with improved insulin signaling [7]. Narath et al. reported the decrease of lactate (Krebs' intermediate cycle) after RYGB. They also observed the higher levels of the high-density lipoprotein and phosphatidylcholine after bariatric surgery [32].

An interesting study about serum uric acid (sUA) was performed by Oberbach et al. An elevated level of sUA was observed in obese patients. However, twelve months after LSG and RYGB, a significant decrease in sUA and other parameters such as BMI, CVD risk factors, hepatic transaminases, and HOMA-IR was observed. Kwon et al. suggested new criteria—7-day metabolomics profile and 3-hydroxybutyrate to glucose ratio for the prediction of 3-month HbA1c. They suggested that this finding could augment current prognostic modalities and help clinicians decide if drug therapy is necessary [37]. Chouiali et al. performed also an interesting but more methodological study. The authors compared two methods (electro-chemiluminescence immunoassay and LC-MS/MS) for measurement of serum 25(OH)D by applying them to the bariatric population [48].

It has to be mentioned that changes presented above are only part of all detected modulations in all investigated studies. They were subjectively chosen by authors as the most interesting. There are still some intriguing modulations which are not described here but can be found in referred articles.

### 4.5. Animal Studies

The animal studies are an important part of the bariatric surgery research. The animal models allow not only to follow the general metabolic changes in blood and urine but also to focus on modulations, in particular organs, by analyses of tissue samples. Five [11, 13, 28, 42, 45] of all presented studies were performed on an animal model. In mentioned studies, not only blood or urine but also liver, heart, and adipose tissue samples were examined. Animal models in this kind of studies are also important because of the fact that they allows to compare particular bariatric procedures between themselves, as well as with sham operations, which cannot be performed in humans. Additionally, the animal models are characterized by high repeatability, which is meaningful when using the metabolomics approach [61].

### 4.6. From Metabolite to Metabolome—General Metabolic Effects of Bariatric Intervention

All above-described alterations in combination with results from MetaboAnalyst showed that bariatric procedures have a huge impact on patients' metabolism. This influence can be observed by following clinical as well as molecular parameters. Investigation of clinical parameters in combination with multiple metabolites provides a broader picture than does evaluation of changes in selected metabolites. Thus, metabolomics is a perfect tool to study global effects evoked by bariatric surgery.

Of course, even the “image” of metabolic changes obtained here, based on all mentioned studies, is still incomplete. There are still some blank areas on this biochemical map. But step by step, using metabolomics techniques (especially combined together) to examine different procedures can bring us more interesting and useful knowledge. Although providing a wide spectrum of information, the metabolomics approach has some limitations. In case of LC-MS, the identification of metabolites could be improved—a relatively large percent of detected metabolites have remained unidentified. In case of GC-MS, identification is based on libraries, so the number of detected metabolites is always limited. NMR in comparison to MS-based platforms is less sensitive. Drawing conclusions is also limited by use of software for pathways analysis. In each software, the number of available metabolites and pathways is limited. MetaboAnalyst library contains 80 biochemical pathways for humans and similar or even less for other species. Therefore, many metabolites and pathways which can be affected by bariatric intervention are not included in such analysis. It is also worthy to collect more data for a particular procedure and to try to analyze them separately. It is very possible that different procedures will evoke distinct impact on biochemical pathways.

## 5. Conclusion

Bariatric procedure strongly influences the metabolism. Detected changes are tied with many compounds and biochemical pathways such as amino acids, lipids, carbohydrates, or gut microbiota alterations. It proves that classification of bariatric intervention as metabolic surgery is appropriate. However, many molecular modulations after those procedures are still unexplained. Therefore, the application of metabolomics in this field of medicine seems to be a right choice. New findings can suggest new directions for surgery technique modifications, contribute to broaden knowledge about obesity and related diseases, and perhaps develop nonsurgical methods of treatment in the future.

## Figures and Tables

**Table 1:** Summary of included studies (in chronological order).

Year	First author	Ref	Metabolomics technique		Sample type		Surgery type	Studied disease	Group size	Time points
2017	Liu, Ruixin	[42]	Untargeted and targeted (amino acids)	HPLC-MS and GC-MS	Human and animal (mice C57BL/6)	Serum	SG	Obesity	29 (23 obese and 6 cases)	Before and one and three months after surgery
2016	Zhao, Linjing	[51]	Targeted (FFA)	UPLC-QTOF MS	Human	Serum	RYGB	Obesity and T2DM	419 individuals (38 obese and diabetic after RYGB and 381 diabetic and nondiabetic with overweight or obesity)	Baseline, 1 year after RYGB
2016	Narath, Sophie H.	[32]	Untargeted	LC-HRMS, HILIC	Human	Serum	RYGB	Obesity	44 patients	2–4 weeks before surgery (PRE), 1–3 weeks after surgery (POST), and one year after surgery follow-up (FU)
2016	Lopes, Thiago I.B.	[33]	Untargeted	^1^H NMR	Human	Plasma	RYGB	Obesity and T2DM	10 subjects	Before and 12 months after surgery
2016	Sarosiek, Konrad	[44]	Untargeted	UHPLC-MS/MS and GC-MS	Human	Serum	SG or full GB	Obesity and T2DM	15 patients (nondiabetic with SG and diabetic with SG or GB)	Baseline (prior to diet/surgery) and 14 and 28 days after surgery)
2016	Jung, Jeeyoun	[45]	Untargeted and targeted	^1^H NMR	Animal (rats, 12-wk-old male Sprague-Dawley)	Serum and liver tissue	DJB	Surgery impact	15 serum (7 sham, 8 DJB), 16 tissue (7 sham, 9 DJB)	No time points, controls versus DJB rats
2016	Chouiali, Ahlem	[48]	Targeted (vit. D)	LC-MS (QQQ)	Human	Serum	BPD	Bariatric population supplemented with vit. D	48 healthy subjects, post-BPD: 44 supplemented with vit. D3 and 30 with vit. D2	During patients follow-up
2016	Nemati, Reza	[30]	Targeted (NEFAs)	LC-MS/MS (QQQ)	Human	Plasma	LGB (Roux), LSG	Obesity and T2DM	38 obese patients—11 GBP, 14 SG, 13 VLCD	Before and 3 days after intervention
2016	García-Alonso, Veronica	[50]	Targeted (eicosanoids)	LC-MS/MS (QQQ)	Human	Omental adipose tissue	“Laparoscopic bariatric surgery”	Regulatory actions of PGs in human omental WAT from obese patients	12 obese individuals after bariatric surgery and 10 patients without obesity after laparoscopic cholecystectomy	Surgery versus controls
2016	Bankoglu, Ezgi Eyluel	[13]	Targeted (8-oxoGua, 8-oxodG, 8-oxoGuo)	LC-MS/MS (QQQ)	Animal (rats, 12-wk-oldmale Zucker)	Urine	RYGB	Obesity, oxidative/nitrative stress, genomic damage	15 RYGB, 17 sham surgery	0 and 27 days
2016	Stratmann, B.	[49]	Targeted (lipoproteins)	^1^H NMR	Human	Serum	DJBL	Morbid obesity and T2DM	18 subjects, finally 16; lipidome for 10 patients	NMR lipidomics baseline and 12 months
2016	Luo, Ping	[25]	Untargeted	UPLC-MS	Human	Serum	RYGB	Obesity and T2DM	35 subjects—23 remission, 12 nonremission of T2DM	At baseline and 6 and 12 months after RYGB
2015	Gralka, Ewa	[38]	Untargeted	^1^H NMR	Human	Serum	SG, proximal or distal RYGB	Obesity	106 obese patients (19 SG, 27 proximal RYGB, 60 distal RYGB), 19 normal weight volunteers, 30 subject with matched BMI	Before and 3, 6, 9, and 12 months after procedures
2015	Bojsen-Moller, Kristine N.	[34]	Targeted (leucine, phenylalanine)	GC-MS	Human	Plasma	RYGB	Obesity	10 obese subject after RYGB (1 out BCZ complications); 9 obese glucose-tolerant	Before and 3 months after surgery
2015	Calvo, Nahum	[40]	Untargeted	^1^H NMR	Human	Hepatic tissue	LSG	Nonalcoholic fatty liver disease (NAFLD)	47 patients, finally included 19	Before (<24 h) and 12 months after surgery
2015	Modesitt, Susan C.	[7]	Untargeted	GC-MS and UPLC-MS/MS	Human (women only)	Serum	LRYGB, LSG, LGB, open RYGB	Endometrial histology, obesity	71 (41 LRYGB, 17 LSG, 8 LGB, 2 open RYGB, 3 no surgery)	Pre- and postoperatively
2015	Arora, Tulika	[36]	Untargeted	GC-MS and UPLC-MS	Human	Plasma	RYGB	Insulin-resistant morbidly obese subjects, some with diabetes	16 patients	Presurgery and 4 and 42 days after surgery
2015	Lopes, Thiago I.B.	[35]	Untargeted and targeted (FA)	^1^H NMR and GC-MS	Human	Plasma	RYGB	Obesity and T2DM	10 patients	Before and 12 months after surgery
2015	Hertel, Johannes	[26]	Untargeted	^1^H NMR	Human	Urine	SG, RYGB	Measurement of biological age based on metabolomics profiles, expanded on clinical samples of obese patients after bariatric surgery	4068 individuals from SHIP-0 at baseline, 996 from SHIP-TREND;38 individuals after surgery	For operated patients: preoperative and postoperative (median follow-up 366.5 days)
2014	Kwon, Hyuk Nam	[37]	Untargeted and targeted (3-HB)	^1^H NMR and GC-MS	Human	Plasma	RYGB, DJB	Patients who underwent surgery for uncontrolled diabetes	22 patients	Metabolic profile 7 days after surgery
2014	Oberbach, Andreas	[31]	Targeted (sUA)	LC-MS/MS	Human	Serum	LSG and RYGB	Obesity	10 severely obese adolescents (5 LSG, 5 RYGB) and 17 normal weight	Pre- and 12 months postoperatively
2014	Kaska, Lukasz	[52]	Targeted (FA)	GC-MS	Human	Serum	Analysis before the surgery	Morbid obesity, nondiabetic	16 women	Samples from the day of surgery
2014	Jüllig, Mia	[27]	Untargeted	GC-MS	Human	Plasma	GB, SG	Subject with T2D undergoing GBP or SG	15 subjects (8 GBP, 7 SG)	3 days before and 3 days after surgery
2014	Heffron, Sean P.	[46]	Targeted	NMR spectroscopy	Human	Fasting blood samples	LGB	Obesity and obesity-relatedcomorbidity	50 obese patients, 47 with completed follow-up	Baseline and 1, 2, 3, 4, and 5 years after operation
2013	Sledzinski, Tomasz	[28]	Targeted (FA)	GC-MS	Human and animal (Rats, 10-wk-old male Wistar)	Serum and adipose tissue	RYGB	Obesity	16 obese nondiabetic women after RYGB, 20 rats (10 controls, 10 diet)	Tissue from women during surgery, rats one month after starting diet
2013	Ashrafian, Hutan	[11]	Untargeted	^1^H NMR and UPLC-MS	Animal (rats, male Wistar)	Plasma and heart tissue extracts	RYGB	Bariatric surgical on cardiac metabolites	27 rats—13 RYGB, 14 sham-operated	8 weeks postoperation
2012	Oberbach, Andreas	[41]	Targeted	LC-MS	Human	Serum	LSG	Morbid obesity (children)	6 obese children	Pre- and 6 months postoperatively
2012	Friedrich, Nele	[39]	Untargeted and targeted	^1^H NMR	Human	Urine	SG, RYGB	Obesity	50 patients (39 SG, 11 RYGB) 50 controls; finally 47 preoperation, 45 postoperation, 48 controls	Pre- and postoperatively (3, 4, 5, 6, 7, and 9 days postsurgery)
2011	Oberbach, Andreas	[43]	Targeted	LC-MS	Human	Serum	SG	Obesity	14 obese after SG, 12 on hypocaloric diet, 17 healthy subjects	Comparison obese subjects and controls, samples six month after treatment
2010	Calvani, R.	[47]	Untargeted	^1^H NMR	Human	Urine	BPD, RYGB	Morbid obesity (insulin resistant)	15 obese subjects, 10 matched controls in general, 2 subjects after bariatric surgery	30 and 90 days after surgery

**Table 2:** The detailed results from the pathway analysis.

Pathway name	*p*	FDR	Impact
Aminoacyl-tRNA biosynthesis	1.0541*E* − 6	5.8083*E* − 5	0.16902
Glycine, serine, and threonine metabolism	1.4521*E* − 6	5.8083*E* − 5	0.48394
Nitrogen metabolism	2.9454*E* − 5	7.8545*E* − 4	6.7*E* − 4
Phenylalanine metabolism	5.8852*E* − 4	0.01177	0.20468
Cysteine and methionine metabolism	9.5617*E* − 4	0.015299	0.54182
Citrate cycle (TCA cycle)	0.0014874	0.015453	0.28353
Taurine and hypotaurine metabolism	0.0014874	0.015453	0.46583
Valine, leucine, and isoleucine biosyntheses	0.0015467	0.015453	0.12084
Propanoate metabolism	0.0017384	0.015453	0.07344
Nicotinate and nicotinamide metabolism	0.0020764	0.016611	0.06485
Alanine, aspartate, and glutamate metabolism	0.0041218	0.029977	0.53182
Arginine and proline metabolism	0.0045157	0.030104	0.4641
Synthesis and degradation of ketone bodies	0.0051467	0.031672	0.7
Pyrimidine metabolism	0.0057644	0.032939	0.22308
Methane metabolism	0.0062792	0.033489	0.18217
Glutathione metabolism	0.011733	0.058666	0.34321
Glyoxylate and dicarboxylate metabolism	0.016707	0.077524	0.1897
Pyruvate metabolism	0.017733	0.077524	0.42654
Purine metabolism	0.018412	0.077524	0.30417
Pantothenate and CoA biosyntheses	0.031274	0.1251	0.0
d-Glutamine and d-glutamate metabolism	0.033063	0.12596	0.35294
Valine, leucine, and isoleucine degradation	0.048233	0.16777	0.0835
Butanoate metabolism	0.048233	0.16777	0.18589
Glycolysis or gluconeogenesis	0.053056	0.17685	0.09576
Linoleic acid metabolism	0.074881	0.23962	0.65625
Cyanoamino acid metabolism	0.087776	0.27008	0.0
Primary bile acid biosynthesis	0.091995	0.27258	0.08068
d-Arginine and d-ornithine metabolism	0.096427	0.27551	0.0
Sulfur metabolism	0.11609	0.31315	0.06614
Glycerophospholipid metabolism	0.11743	0.31315	0.17061
Galactose metabolism	0.13757	0.35503	0.24385
Vitamin B6 metabolism	0.16454	0.39889	0.24174
Glycerolipid metabolism	0.16454	0.39889	0.27975
Thiamine metabolism	0.21633	0.50901	0.0
Fatty acid biosynthesis	0.23092	0.52123	0.0
Sphingolipid metabolism	0.23455	0.52123	0.04244
Tyrosine metabolism	0.24772	0.5356	0.12506
Phenylalanine, tyrosine, and tryptophan biosyntheses	0.27174	0.57208	0.008
*beta*-Alanine metabolism	0.29058	0.59605	0.0
*alpha*-Linolenic acid metabolism	0.30951	0.61902	0.20335
Histidine metabolism	0.34348	0.67021	0.21313
Selenoamino acid metabolism	0.44198	0.84186	0.00321
Biotin metabolism	0.5361	0.9974	0.0
Ascorbate and aldarate metabolism	0.59325	1.0	0.13047
Arachidonic acid metabolism	0.60998	1.0	0.2595
Lysine degradation	0.62333	1.0	0.14675
Glycosylphosphatidylinositol- (GPI-) anchor biosynthesis	0.62401	1.0	0.0
Tryptophan metabolism	0.62432	1.0	0.24863
Pentose phosphate pathway	0.64591	1.0	0.0
Lysine biosynthesis	0.64591	1.0	0.09993
Starch and sucrose metabolism	0.66555	1.0	0.0765
Pentose and glucuronate interconversions	0.70426	1.0	0.02401
Porphyrin and chlorophyll metabolism	0.71374	1.0	0.05249
Inositol phosphate metabolism	0.75054	1.0	0.18387
Riboflavin metabolism	0.76996	1.0	0.0
Caffeine metabolism	0.76996	1.0	0.0305
Fatty acid elongation in mitochondria	0.84921	1.0	0.0
Terpenoid backbone biosynthesis	0.90126	1.0	0.0
Ubiquinone and other terpenoid-quinone biosyntheses	0.92013	1.0	0.0
Amino sugar and nucleotide sugar metabolism	0.9439	1.0	0.0
Fructose and mannose metabolism	0.96591	1.0	0.0
Steroid hormone biosynthesis	0.96878	1.0	0.10049
Fatty acid metabolism	0.97043	1.0	0.02959

Raw *p* is the original *p* value calculated from the enrichment analysis; the FDR *p* is the *p* value adjusted using false discovery rate; the Impact is the pathway impact value calculated from pathway topology analysis.
